# Supplementary material for: Applications of Wearable Technology in a Real-Life Setting in People with Knee Osteoarthritis: A Systematic Scoping Review
Source: J Clin Med. 2021 Nov 30;10(23):5645. doi: 10.3390/jcm10235645 (PMC8658504; doi:10.3390/jcm10235645)
Supplement: Supplementary file 1 [file jcm-10-05645-s001.zip › jcm-1448687-supplementary/jcm-1448687-supplementary.pdf]

Table S1. Preferred reporting items for systematic reviews and meta-analyses extension for scoping reviews (PRISMA-ScR) checklist.

Section	Item	PRISMA-ScR Checklist Item	Reported on page
		Title	
**Title**	1	Identify the report as a scoping review.	1
		Abstract	
**Structured summary**	2	Provide a structured summary that includes (as applicable): background, objectives, eligibility criteria, sources of evidence, charting methods, results, and conclusions that relate to the review questions and objectives.	1
		Introduction	
**Rationale**	3	Describe the rationale for the review in the context of what is already known. Explain why the review questions/objectives lend themselves to a scoping review approach.	1–2
**Objectives**	4	Provide an explicit statement of the questions and objectives being addressed with reference to their key elements (e.g., population or participants, concepts, and context) or other relevant key elements used to conceptualize the review questions and/or objectives.	2
		Methods	
**Protocol and registration**	5	Indicate whether a review protocol exists; state if and where it can be accessed (e.g., a Web address); and if available, provide registration information, including the registration number.	2
**Eligibility criteria**	6	Specify characteristics of the sources of evidence used as eligibility criteria (e.g., years considered, language, and publication status), and provide a rationale.	3
**Information sources***	7	Describe all information sources in the search (e.g., databases with dates of coverage and contact with authors to identify additional sources), as well as the date the most recent search was executed.	3
**Search**	8	Present the full electronic search strategy for at least one database, including any limits used, such that it could be repeated.	Appendix B
**Selection of sources of evidence†**	9	State the process for selecting sources of evidence (i.e., screening and eligibility) included in the scoping review.	3–4
**Data charting process‡**	10	Describe the methods of charting data from the included sources of evidence (e.g., calibrated forms or forms that have been tested by the team before their use, and whether data charting was done independently or in duplicate) and any processes for obtaining and confirming data from investigators.	4
**Data items**	11	List and define all variables for which data were sought and any assumptions and simplifications made.	Appendix C
**Critical appraisal of individual sources of evidence§**	12	If done, provide a rationale for conducting a critical appraisal of included sources of evidence; describe the methods used and how this information was used in any data synthesis (if appropriate).	NA
**Synthesis of results**	13	Describe the methods of handling and summarizing the data that were charted.	4
		Results	
**Selection of sources of evidence**	14	Give numbers of sources of evidence screened, assessed for eligibility, and included in the review, with reasons for exclusions at each stage, ideally using a flow diagram.	4
**Characteristics of sources of evidence**	15	For each source of evidence, present characteristics for which data were charted and provide the citations.	4–10

**Critical appraisal within sources of evidence**	16	If done, present data on critical appraisal of included sources of evidence (see item 12).	NA
**Results of individual sources of evidence**	17	For each included source of evidence, present the relevant data that were charted that relate to the review questions and objectives.	4–10
**Synthesis of results**	18	Summarize and/or present the charting results as they relate to the review questions and objectives.	4–10
		Discussion	
**Summary of evidence**	19	Summarize the main results (including an overview of concepts, themes, and types of evidence available), link to the review questions and objectives, and consider the relevance to key groups.	10-14
**Limitations**	20	Discuss the limitations of the scoping review process.	14
**Conclusions**	21	Provide a general interpretation of the results with respect to the review questions and objectives, as well as potential implications and/or next steps.	15
		Funding	
**Funding**	22	Describe sources of funding for the included sources of evidence, as well as sources of funding for the scoping review. Describe the role of the funders of the scoping review.	15

Table S2. Search strategies for each search engines.

**Database**	**Date**	**N**	**Search terms**
			Primary search
MEDLINE (Pubmed)	03/06/21	804	(knee[MeSH Terms] OR knee joint[MeSH Terms] OR knee[Title/Abstract] OR tibiofemoral[Title/Abstract]) OR patellofemoral[Title/Abstract]) AND (osteoarthritis[MeSH Terms] OR osteoarthrosis[MeSH Terms] OR joint disease[MeSH Terms] OR arthritis[MeSH Terms] OR osteoarthrit*[Title/Abstract] OR osteoarthros*[Title/Abstract] OR degenerative arthr*[Title/Abstract] OR arthros*[Title/Abstract] OR arthrit*[Title/Abstract] OR gonarthr*[Title/Abstract]) AND (wearable device[Title/Abstract] OR wearable sensor[Title/Abstract] OR electronic skin wearable technology[Title/Abstract] OR wearable electronic devices[Title/Abstract] OR smart watch[Title/Abstract] OR smartwatch[Title/Abstract] OR IMU[Title/Abstract] OR inertial measurement unit[Title/Abstract] OR accelerometery[Title/Abstract] OR accelerometer[Title/Abstract] OR gyroscope[Title/Abstract] OR ambulatory[Title/Abstract] OR free living[Title/Abstract] OR in field[Title/Abstract] OR ambulatory monitoring[Title/Abstract] OR real world[Title/Abstract] OR in the wild[Title/Abstract] OR telehealth[Title/Abstract] OR smartwatch app[Title/Abstract] OR mobile app[Title/Abstract] OR pressure insoles[Title/Abstract] OR e-health[Title/Abstract])
CINAHL	03/06/21	442	(AB knee OR AB tibiofemoral OR AB patellofemoral) AND (AB osteoarthritis OR AB osteoarthrosis OR AB arthritis OR AB arthrosis OR AB "joint disease" OR AB gonarthr*) AND (AB "wearable device" OR AB "wearable sensor" OR AB "electronic skin wearable technology" OR AB "wearable electronic devices" OR AB "smart watch" OR AB smartwatch OR AB "inertial measurement unit" OR AB accelerometery OR AB accelerometer OR AB gyroscope OR AB ambulatory OR AB "free living" OR AB "in field" OR AB "ambulatory monitoring" OR AB "real world" OR AB "in the wild" OR AB telehealth OR AB "smartwatch application" OR AB "mobile application" OR AB "pressure insoles" OR AB e-health)
SCOPUS	03/06/21	513	TITLE-ABS (knee OR tibiofemoral OR patellofemoral) AND TITLE-ABS (osteoarthritis OR osteoarthrosis OR "joint disease" OR arthritis OR arthrosis OR "degenerative joint disease" OR gonarthr*) AND TITLE-ABS ("wearable device" OR "wearable sensor" OR "electronic skin wearable technology" OR "wearable electronic devices" OR "smart watch" OR "smartwatch" OR "inertial measurement unit" OR accelerometery OR accelerometer OR gyroscope OR ambulatory OR "free living" OR "in field" OR "ambulatory monitoring" OR "real world" OR "in the wild" OR telehealth OR "smartwatch application" OR "mobile application" OR "pressure insoles" OR e-health)
WEB OF SCIENCE	03/06/21	372	AB = (((knee OR tibiofemoral OR patellofemoral) AND (osteoarthritis OR osteoarthrosis OR "joint disease" OR arthritis OR arthrosis OR "degenerative joint disease" OR gonarthr*) AND ("wearable device" OR "wearable sensor" OR "electronic skin wearable technology" OR "wearable electronic devices" OR "smart watch" OR "smartwatch" OR "inertial measurement unit" OR accelerometery OR accelerometer OR gyroscope OR ambulatory OR "free living" OR "in field" OR "ambulatory monitoring" OR "real world" OR "in the wild" OR telehealth OR "smartwatch application" OR "mobile application" OR "pressure insoles" OR e-health))) Indexes=SCI-EXPANDED, SSCI, A&HCI, CPCI-S, CPCI-SSH, ESCI Timespan=All years

Database	Date	N	Search terms
EMBASE	03/06/21	1182	((knee or tibiofemoral or patellofemoral) and (osteoarthritis or osteoarthrosis or arthritis or arthrosis or gonarthritis or gonarthrosis) and (wearable or sensor or smartwatch or inertial measurement unit or accelerometery or accelerometer or gyroscope or telehealth or mobile or insole or e-health)).ti,ab
IEEE Digital Library	03/06/21	110	(((knee OR tibiofemoral OR patellofemoral) AND (osteoarthritis OR osteoarthrosis OR arthritis OR arthrosis OR joint disease OR degenerative joint disease OR gonarthr*) AND (wearable device OR wearable sensor OR electronic skin wearable technology OR wearable electronic device OR smart watch OR smartwatch OR inertial measurement unit OR accelerometery OR accelerometer OR gyroscope OR ambulatory OR free living OR in field OR ambulatory monitoring OR real world OR in the wild OR telehealth OR smartwatch application OR mobile application OR pressure insoles OR e-health)))
COCHRANE Library	03/06/21	182	("knee joint") OR (tibiofemoral) OR ("patello femoral") in Title Abstract Keyword AND ("osteoarthritis") OR ("osteoarthrosis") OR ("arthritis") OR ("arthritic") in Title Abstract Keyword AND (wearable device) OR (wearable sensor) OR ("electronic skin wearable technology") OR ("wearable electronic device") OR (smart watch) OR (smartwatch) OR ("inertial measurement unit") OR (accelerometery) OR (accelerometer) OR (gyroscope) OR (ambulatory) OR ("free living") OR ("in field") OR ("ambulatory monitoring") OR ("real world") OR ("in the wild") OR (telehealth) OR ("smartwatch application") OR ("mobile application") OR (pressure insoles) OR (e-health) in Title Abstract Keyword
Secondary search			
ACM Digital Library	03/06/21	3	[[Abstract: knee] OR [Abstract: tibiofemoral] OR [Abstract: patellofemoral]] AND [[Abstract: osteoarthritis] OR [Abstract: osteoarthrosis] OR [Abstract: "joint disease"] OR [Abstract: arthritis] OR [Abstract: arthrosis] OR [Abstract: "degenerative joint disease"] OR [Abstract: gonarthr*]] AND [[Abstract: "wearable device"] OR [Abstract: "wearable sensor"] OR [Abstract: "electronic skin wearable technology"] OR [Abstract: "wearable electronic devices"] OR [Abstract: "smart watch"] OR [Abstract: "smartwatch"] OR [Abstract: "inertial measurement unit"] OR [Abstract: accelerometery] OR [Abstract: accelerometer] OR [Abstract: gyroscope] OR [Abstract: ambulatory] OR [Abstract: "free living"] OR [Abstract: "in field"] OR [Abstract: "ambulatory monitoring"] OR [Abstract: "real world"] OR [Abstract: "in the wild"] OR [Abstract: telehealth] OR [Abstract: "smartwatch application"] OR [Abstract: "mobile application"] OR [Abstract: "pressure insoles"] OR [Abstract: e-health]]
Google Scholar	03/06/21	35	allintitle: knee AND osteoarthritis AND "wearable device" OR "wearable sensor" OR "wearable technology" OR "wearable electronic devices" OR "smart watch" OR smartwatch OR "inertial measurement unit" OR accelerometer OR ambulatory OR "free living"
Reference lists	21/07/21	13	NA

Table S3. Data extraction template.

Data items	Associated questions
**Publication details**	
Author	What was the name of the first author?
Year	When was the study published?
Title	What was the title of the publication?
Affiliations	What is the affiliation of the first author?
Type of evidence	In what type of literature was the study published?
Language	Which language is the study published?
Funding source	Who funded the study?
Role of the funder	What was the role of the funder in the study?
**General study details**	
Study design	What was the study's design?
Country	In which country(s) did the study take place?
Study aim(s)	List study's aims
Population	What was the studied population?
Study size	How many people with knee osteoarthritis (OA) participated in the study?
Osteoarthritis diagnosis	How was OA diagnosis confirmed?
Age	What was the mean age of study participants?
Ethnicity	What was the ethnicity of the studied population?
Sex	How many females constituted the study group?
Body mass index	What was the mean BMI of study participants?
**Research question 1** - What type of wearable technology is utilized in a real-world setting in people with knee OA?	
Wearable technology type	What was the type of wearable technology used in the study?
Brand and model	What was the brand and model of wearable technology?
Number of sensors	How many sensors does the wearable has?

Size	What was the size of wearable device (length, high, width)?
Weight	What was the weight of wearable device?
Range	What range was the data collected at with wearable technology?
Sampling frequency	What was the sampling frequency the data was collected with?
Placement form	How was the wearable device used i.e., attached to body, portable?
**Research question 2** - What are the applications of wearable technology in a real-world setting in people with knee OA?	
Aim of use	What were the applications of wearable technology?
Type of data	If applicable, what type of data was wearable device measuring?
Data metric	Which specific metrics authors extracted from data obtained from wearable device?
Duration of use	For how long participants used a wearable device?
Task description form	How were instructions for using a wearable device provided to participants?
Placement location	To which part of the body was wearable device attached to?
**Research question 3 -** What are the facilitators to feasibility of using wearable technology in a real-world setting in people with knee OA?	
Facilitators to feasibility	What facilitators of using wearable technology in a real-world setting did authors describe?
Limitations	What limitations of wearable technology did authors describe?
Public involvement	Were participants involved in research, excluding being sole research participants?
Validity of technology	Did authors report on validity of device? If yes, which population?
Reliability of technology	Did authors report on reliability of device? If yes, which population?
Acceptability	Did authors evaluate acceptability of a wearable device?
Adverse events	Did authors evaluate adverse events related to using a wearable device? If yes, which adverse events they listed?
Adherence	Did author report adherence to using wearable device?
Code	Did author include code for analysis of data from a wearable device?
Data	Did authors include raw or semi-processed wearable tech data with the publication?
Price	Did authors report price of wearable technology
Battery	Did authors report battery life duration? If yes, what was it?
Data storage capacity	Did authors report data storage capacity of a wearable device? If yes, what was it?
Data transfer	Did authors report how is data from a wearable transferred? If yes, what was it?
Data analysis methods	Did authors report how they analysed sensor data obtained from a wearable device?
Water resistant	Did authors report whether wearable device is water resistance?
